# Histopathological Validation of Dark‐Blood Late Gadolinium Enhancement MRI Without Additional Magnetization Preparation

**DOI:** 10.1002/jmri.27805

**Published:** 2021-06-24

**Authors:** Robert J. Holtackers, Suzanne Gommers, Luuk I.B. Heckman, Caroline M. Van De Heyning, Amedeo Chiribiri, Frits W. Prinzen

**Affiliations:** ^1^ Cardiovascular Research Institute Maastricht (CARIM) Maastricht University Maastricht The Netherlands; ^2^ Department of Radiology and Nuclear Medicine Maastricht University Medical Centre Maastricht The Netherlands; ^3^ School of Biomedical Engineering & Imaging Sciences King's College London London UK; ^4^ Department of Physiology Maastricht University Maastricht The Netherlands; ^5^ Department of Cardiology Antwerp University Hospital Edegem Belgium

**Keywords:** myocardial infarction, cardiac magnetic resonance, magnetic resonance imaging, late gadolinium enhancement, dark‐blood LGE, histology

## Abstract

**Background:**

Conventional bright‐blood late gadolinium enhancement (LGE) cardiac magnetic resonance imaging (MRI) often suffers from poor scar‐to‐blood contrast due to the bright blood pool adjacent to the enhanced scar tissue. Recently, a dark‐blood LGE method was developed which increases scar‐to‐blood contrast without using additional magnetization preparation.

**Purpose:**

We aim to histopathologically validate this dark‐blood LGE method in a porcine animal model with induced myocardial infarction (MI).

**Study Type:**

Prospective.

**Animal Model:**

Thirteen female Yorkshire pigs.

**Field Strength/Sequence:**

1.5 T, two‐dimensional phase‐sensitive inversion‐recovery radiofrequency‐spoiled turbo field‐echo.

**Assessment:**

MI was experimentally induced by transient coronary artery occlusion. At 1‐week and 7‐week post‐infarction, in‐vivo cardiac MRI was performed including conventional bright‐blood and novel dark‐blood LGE. Following the second MRI examination, the animals were sacrificed, and histopathology was obtained. Matching LGE slices and histopathology samples were selected based on anatomical landmarks. Independent observers, while blinded to other data, manually delineated the endocardial, epicardial, and infarct borders on either LGE images or histopathology samples. The percentage of infarcted left‐ventricular myocardium was calculated for both LGE methods on a per‐slice basis, and compared with histopathology as reference standard. Contrast‐to‐noise ratios were calculated for both LGE methods at 1‐week and 7‐week post‐infarction.

**Statistical Tests:**

Pearson's correlation coefficient and paired‐sample *t*‐tests were used. Significance was set at *P* < 0.05.

**Results:**

A combined total of 24 matched LGE and histopathology slices were available for histopathological validation. Dark‐blood LGE demonstrated a high level of agreement compared to histopathology with no significant bias (−0.03%, *P* = 0.75). In contrast, bright‐blood LGE showed a significant bias of −1.57% (*P* = 0.03) with larger 95% limits of agreement than dark‐blood LGE. Image analysis demonstrated significantly higher scar‐to‐blood contrast for dark‐blood LGE compared to bright‐blood LGE, at both 1‐week and 7‐weeks post‐infarction.

**Data Conclusion:**

Dark‐blood LGE without additional magnetization preparation provides superior visualization and quantification of ischemic scar compared to the current in vivo reference standard.

**Level of Evidence:**

1

**Technical Efficacy Stage:**

2

For already two decades, late gadolinium enhancement (LGE) cardiac magnetic resonance imaging (MRI) has been considered the method of choice for the non‐invasive assessment of myocardial viability.^1^ The well‐established bright‐blood inversion‐recovery LGE sequence can distinguish areas of necrosis and macroscopic scarring from normal myocardium,^2^, ^3^ and forms the basis of clinical routine cardiac MRI protocols worldwide. A major drawback of conventional bright‐blood LGE, however, is the poor contrast between scar tissue and the blood pool. Since both tissues may have similar high‐intensity signal, determining the scar‐blood barrier is often challenging. In cases with subendocardial scar or papillary muscle scar, the apparent scar volume can be substantially underestimated or even completely obscured.^4^

In the past 15 years, various dark‐ and black‐blood LGE methods were introduced aiming to increase the scar‐to‐blood contrast and thereby improve subendocardial scar conspicuity.^5^ The majority of these methods use additional magnetization preparation mechanisms, such as T_2_ preparation,^6^, ^7^, ^8^, ^9^, ^10^, ^11^ magnetization transfer,^12^, ^13^ spin‐locking,^13^ and repetitive inversion pulses,^14^, ^15^ to achieve this. Implementing these additional preparation mechanisms into a standard LGE sequence, however, requires scanner software modifications and thereby inherently hinders the widespread clinical availability of these methods. In 2017, a novel LGE method that suppresses the blood‐pool signal without using additional magnetization preparation was introduced.^16^ This readily available dark‐blood LGE approach proved its superiority in ischemic scar detection, observer confidence, and overall image quality, compared to conventional LGE, in a large, unselected cohort of 300 patients.^17^ However, no histopathology was available as reference standard.

In the present study, we aimed to histopathologically validate this novel dark‐blood LGE approach in a porcine animal model with induced myocardial infarction (MI).

## Materials and Methods

**FIGURE 1:**
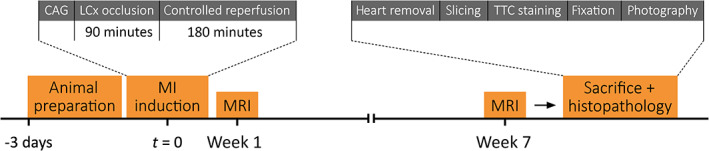
Study overview and timeline. CAG = coronary angiography; LCx = circumflex branch of the left coronary artery; MI = myocardial infarction; MRI = magnetic resonance imaging; TTC = triphenyltetrazolium chloride.

This study involved in‐vivo cardiac MRI evaluation and histopathological analysis of reperfused experimentally induced MI caused by transient coronary artery occlusion in 13 female 15‐weeks‐old Yorkshire pigs (Fig. 1). Infarct size as measured by dark‐blood LGE and conventional bright‐blood LGE were compared with histopathology as reference standard. Additionally, contrast‐to‐noise ratios (CNRs) were assessed for both dark‐blood and conventional bright‐blood LGE. Animal handling was performed according to the Dutch Law on Animal Experimentation and the European Directive on the Protection of Animals used for Scientific Purposes (2010/63/EU). The protocol was approved by the Experimental Animal Committee of the Maastricht University (DEC2016‐002).

### Animal Preparation

Three days prior to MI induction, the animals received daily prophylactic treatment with 200 mg amiodarone, orally administered. Animals were kept fasting for 18 hours before the experiment and were premedicated with zoletil (5–8 mg/kg intramuscularly). After thiopental induction (5–15 mg/kg intravenously), anesthesia was maintained by continuous intravenous infusion of propofol (2.5–10 mg/kg/hour), sufentanil (4–8 mg/kg/hour), and rocuronium (0.1 mg/kg/hour). A thermal mattress was used to maintain adequate body temperature. Electrocardiograms were derived from the limb leads.

Following catheterization of the left coronary artery ostium, a left coronary angiogram was made using diluted contrast agent. Through the guiding catheter, a guidewire was introduced in the left circumflex artery (LCx). An over‐the‐wire balloon catheter was then advanced into the LCx, leaving the first visible branch on fluoroscopy free from occlusion. The remainder of the LCx was then occluded for 90 minutes, followed by 180 minutes of controlled reperfusion (under anesthesia).

### MRI Protocol

One and 7 week(s) after MI induction, the animals underwent cardiac MRI examination under general anesthesia (as during MI induction) using a clinical 1.5 T MR system (Ingenia, Philips Healthcare, Best, The Netherlands). Real‐time imaging was performed first to facilitate planning of the stack of short‐axis view and three long‐axis views (left two‐chamber, left‐ventricular (LV) outflow tract, and four‐chamber). Cine images were subsequently acquired in all cardiac views to obtain cardiac volumes and function. An intravenous injection of 0.2 mmol/kg gadobutrol (Gadovist, Bayer Pharmaceuticals, Germany) and subsequent 25 mL saline flush was then administered for LGE imaging. At approximately 10 minutes post‐injection, both conventional bright‐blood LGE and dark‐blood LGE images were acquired in a stack of short‐axis views as well as in the three apical views. Typical parameters for this electrocardiogram‐triggered phase‐sensitive inversion‐recovery (PSIR) sequence with radiofrequency‐spoiled turbo field‐echo (T_1_‐TFE) readout were: echo time (TE) 3.0 msec, repetition time (TR) 6.1 msec, flip angle 25°, PSIR reference flip angle 5°, 22 lines acquired every other RR‐interval, acquired resolution 1.6 × 1.6 mm^2^, reconstructed resolution 0.8 × 0.8 mm^2^, slice thickness 8 mm, sensitivity encoding (SENSE) factor 1.5, two averages. A 1:1 randomization scheme was used to decide the order of acquisition of the two LGE methods. A preceding Look‐Locker sequence was performed for each LGE method to obtain the correct inversion time (TI). For conventional LGE, the TI was set for myocardium nulling, while for dark‐blood LGE the TI was set for LV blood pool nulling. Other settings were identical. The dark‐blood LGE mechanism without using additional magnetization preparation has been described in detail in earlier work.^16^ Additionally, for each LGE method, a dedicated noise scan (identical pulse sequence without excitation pulses) was performed to assess the true noise level. All LGE and noise scan images were acquired in the mid‐diastolic resting period during breath‐holds (ventilator stop). Six weeks later, at 7‐weeks post‐MI, a second MRI examination was performed using an identical scan protocol and maintaining the same LGE scan order as at week 1.

### Histopathology Protocol

Directly following the second MRI examination at 7‐weeks post‐MI, the animals were sacrificed, while still being anesthetized, by exsanguination through removal of the heart. The removed heart was cut into 8 mm thick transverse slices using a three‐dimensional printed mold with equally spaced cutting slots. Each slice was then incubated in 1% triphenyl‐tetrazolium chloride (TTC) for 20 minutes at 37 °C followed by fixation in 4% formalin for 20 minutes at room temperature. While positioned on graph paper, digital photographs were taken from both the apical and basal side of each slice.

### MR Image Analysis

Before analysis of the LGE images and histopathology samples, matching sets were selected based on anatomical landmarks. All LGE images were analyzed for enhanced infarct tissue using a commercially available software package (CAAS MR Solutions 5.2.1, Pie Medical Imaging, Maastricht, The Netherlands). An expert observer with 10+ years of experience in cardiac MRI (SG), who was blinded to histopathology results, manually delineated the endocardial, epicardial, and infarct borders. Papillary muscles and LV trabeculations were excluded from the myocardial contours. For each LGE image containing infarct tissue, the percentage of infarcted LV myocardium was calculated by dividing the infarcted area by the total LV area of that respective slice.

### Histopathology Analysis

The digital photographs of the histopathology samples were analyzed using a freely available software package (ImageJ, U.S. National Institutes of Health, Bethesda, MD, USA). After obtaining correct scaling parameters using the graph paper, the endocardial, epicardial, and infarct borders were manually delineated by another observer, who was blinded to the MRI results (LIBH). Similar to the LGE image analysis, papillary muscles and LV trabeculations were excluded from the myocardial contours. For each histopathology sample containing infarct tissue, the percentage of infarcted LV myocardium was calculated by dividing the infarcted area by the total LV area of that respective sample.

### CNR Analysis

For the CNR analysis, only LGE slices that showed infarcted myocardium for both LGE methods were considered. Applied scaling factors in the image data were removed by converting the data to floating point values, as these reflect the true MR signal range directly after reconstruction.^18^ A custom‐made MATLAB (version 2018b, The MathWorks, Natick, MA, USA) software tool was used to manually draw regions of interest (ROIs) in the infarcted myocardium, normal myocardium, and LV blood pool. For each ROI, the mean signal intensity was calculated as a measure of signal level. A fourth ROI was drawn around the epicardial border of the LV and projected on the corresponding dedicated noise image. The SD of the noise intensity within this ROI was calculated as a measure of LV noise level. For each image, the signal‐to‐noise ratio (SNR) of normal myocardium, LV blood pool, and infarct tissue were then calculated by dividing its signal level by the LV noise level. The scar‐to‐blood, scar‐to‐myocardium, and blood‐to‐myocardium CNRs were calculated by subtracting the SNRs of the two corresponding tissues.

### Statistical Analysis

All statistical analyses were performed using the Statistical Package for the Social Sciences (SPSS, version 26, International Business Machines, Armonk, NY, USA). Results are expressed as mean ± SD or as percentage unless specified otherwise. Correlations between both LGE methods (for week 1 MRI), and between each LGE method (week 7 MRI) and histopathology, for assessing the percentage of infarcted LV myocardium, were evaluated using a Pearson correlation test. Bland–Altman analysis was used to evaluate potential biases and overall agreement in infarct size as assessed by both LGE methods (week 1 MRI), and either LGE method (week 7 MRI) with histopathology as reference standard. Differences in CNRs between both LGE methods (for both week 1 and week 7 MRI) were evaluated using either the paired‐sample *t*‐test (normally distributed data) or the non‐parametric Wilcoxon signed‐rank test (non‐normally distributed data). Normality of data was evaluated using the Shapiro–Wilk test. All statistical tests were two‐tailed and *P*‐values <0.05 were considered significant.

## Results

Five pigs successfully underwent transient coronary artery occlusion and the final MRI examination 7‐week post‐MI, and were sacrificed subsequently for histopathological analysis. From the remaining eight pigs, five died during or within 4 hours after MI induction, two died in a later stage but prior to final MRI examination, and a single pig could not undergo MRI examination due to coronavirus disease 2019 restrictions on the experiments. Animal weight at the MRI examinations 1‐week and 7‐weeks post‐MI was 55.0 ± 11.8 kg and 87.2 ± 8.4 kg, respectively. The TIs used for conventional bright‐blood LGE and dark‐blood LGE were 307 ± 26 msec and 189 ± 25 msec when performed first at approximately 10 minutes post‐injection, and 326 ± 20 msec and 224 ± 13 msec when performed second at approximately 20 minutes post‐injection, respectively.

### Infarct Size

#### ONE‐WEEK POST‐MI

**FIGURE 2:**
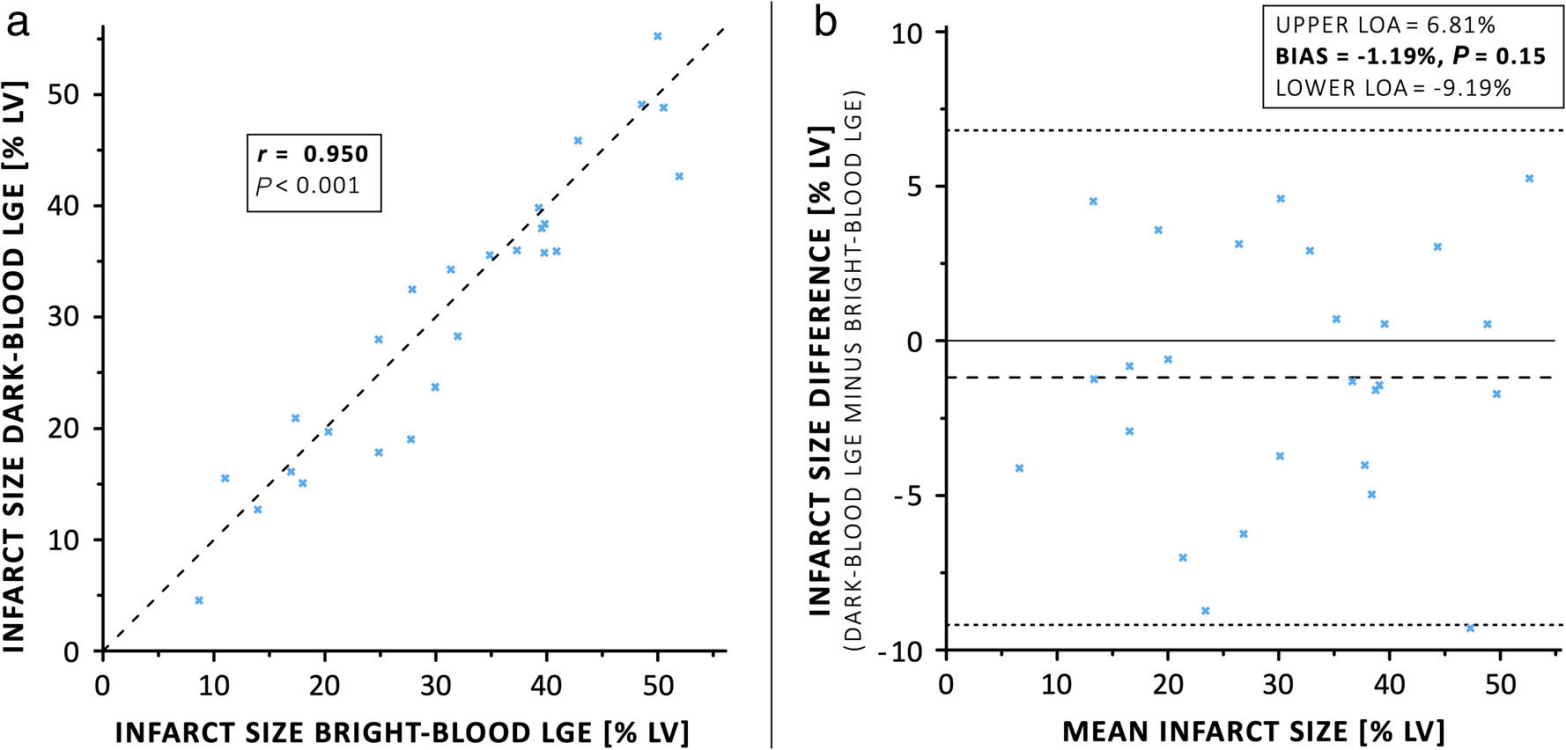
(**a**) Linear regression and (**b**) Bland–Altman plot of infarct size as assessed by conventional bright‐blood LGE and dark‐blood LGE in all slices with infarcted tissue at 1‐week post‐MI. LGE = late gadolinium enhancement; LOA = limits of agreement; LV = left‐ventricular; MI = myocardial infarction.

A total of 26 MRI slices with infarcted regions on both conventional bright‐blood LGE and dark‐blood LGE were acquired 1‐week post‐MI. Infarct size by dark‐blood LGE correlated well with conventional bright‐blood LGE (*r* = 0.950, *P* < 0.001, Fig. 2a) with no significant bias (−1.2%, *P* = 0.15) between both methods by Bland–Altman analysis (Fig. 2b).

#### SEVEN‐WEEKS POST‐MI + HISTOPATHOLOGICAL VALIDATION

**FIGURE 3:**
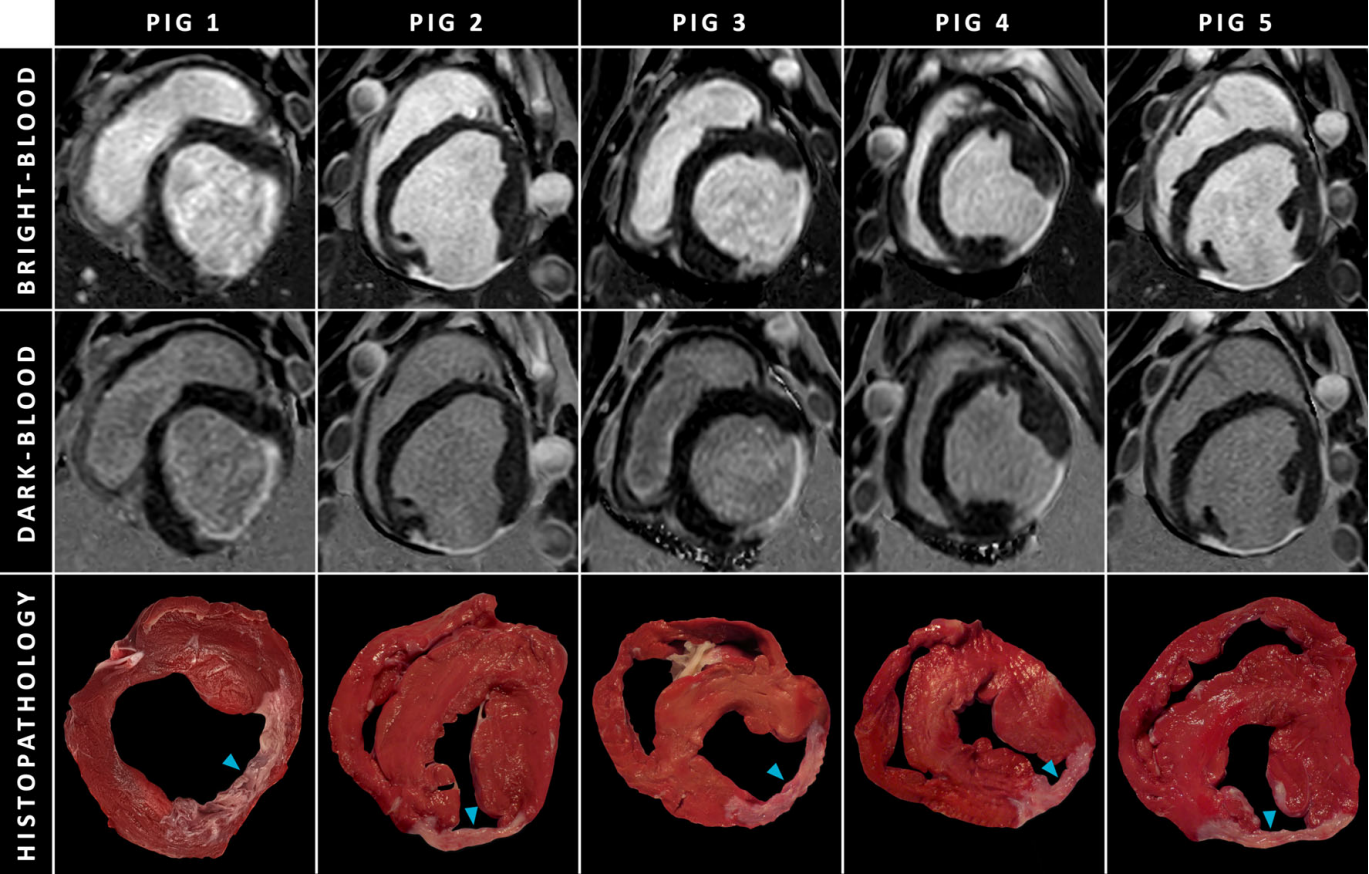
Matching slices of conventional bright‐blood LGE, dark‐blood LGE, and histopathology samples for all pigs. The cyan arrowheads in the histopathology samples indicate the infarct regions. For dark‐blood LGE the inversion time was shortened to the point of blood pool nulling. Although the nulled blood pool appears black in the magnitude image (not displayed), it appears dark gray in the PSIR image as normal myocardium has even lower (negative) magnetization due to its longer T_1_ relaxation time. The negative magnetization of normal myocardium, which would appear bright in a magnitude image (not displayed), appears completely black as PSIR reveals its negative phase. LGE = late gadolinium enhancement; PSIR = phase‐sensitive inversion‐recovery.

**FIGURE 4:**
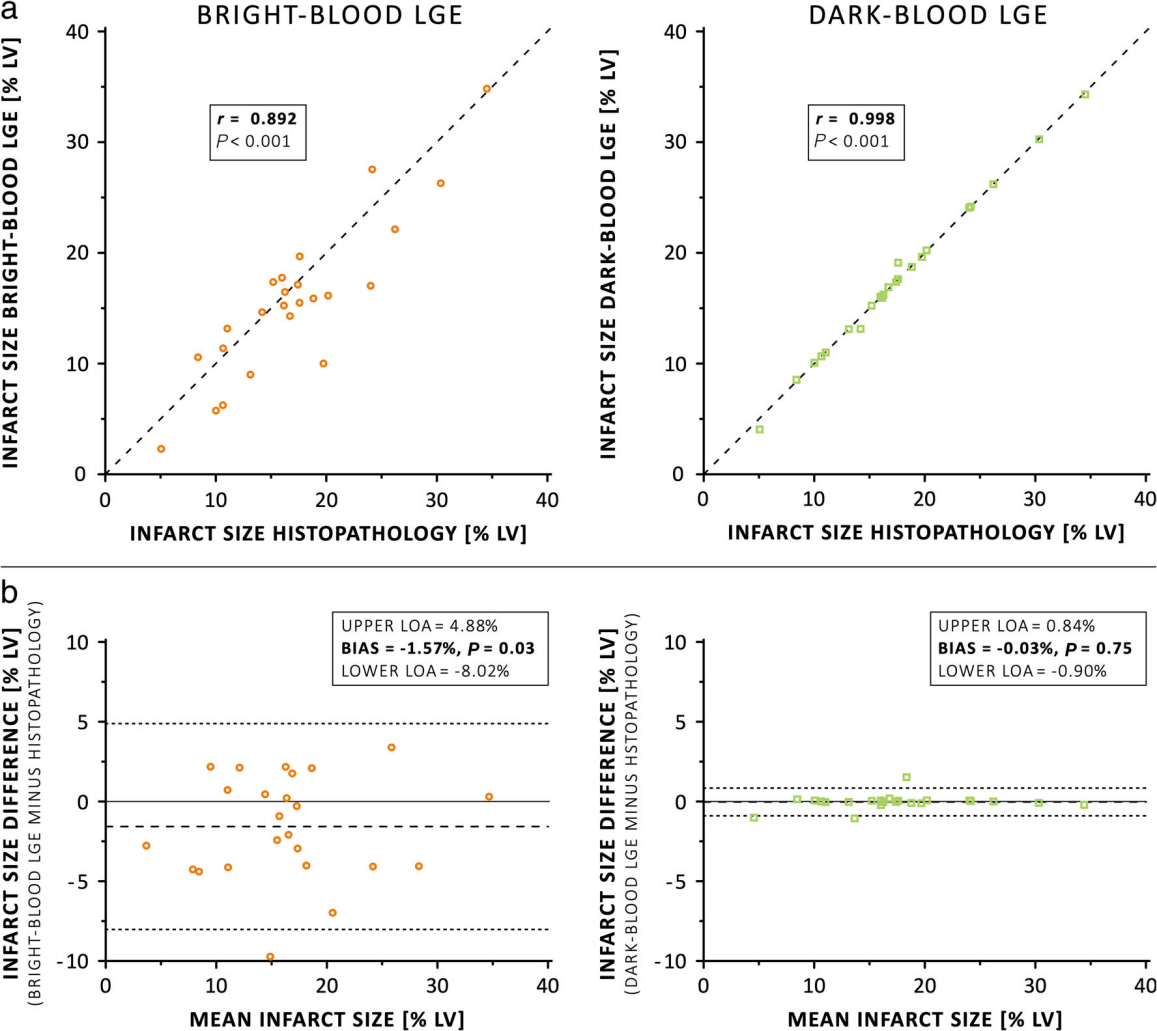
(**a**) Linear regression and (**b**) Bland–Altman plots of infarct size as assessed by both LGE methods with histopathology as reference standard. For both plot types, the results of conventional bright‐blood and dark‐blood LGE are illustrated using red circles and green squares, respectively. Dark‐blood LGE shows higher correlation and better agreement with histopathology compared to conventional bright‐blood LGE. LGE = late gadolinium enhancement; LOA = limits of agreement; LV = left‐ventricular.

**FIGURE 5:**
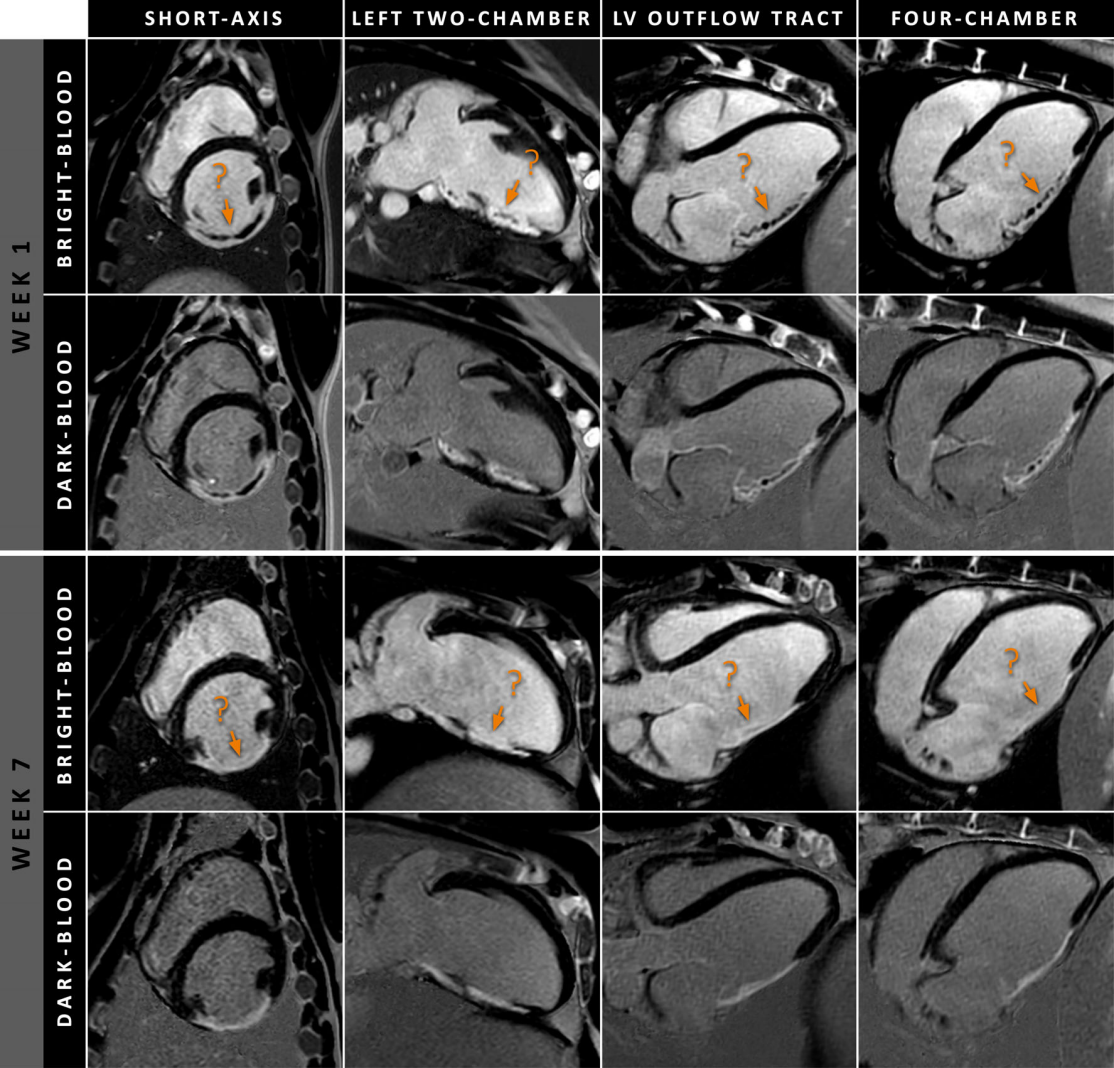
Conventional bright‐blood and dark‐blood LGE images acquired in multiple cardiac views at 1‐week and 7‐weeks post‐MI. The orange arrows indicate the endocardial borders, which are poorly visualized using conventional bright‐blood LGE (orange arrows). In contrast, the same regions are clearly visualized using dark‐blood LGE, enabling an accurate delineation of the endocardial border. Note the areas of microvascular obstruction and/or hemorrhage, appearing as dark cores within the scar areas at 1‐week post‐MI. As bright‐blood LGE was performed before dark‐blood LGE in this subject, these areas appear larger on the bright‐blood LGE images. LGE = late gadolinium enhancement; MI = myocardial infarction.

A combined total of 24 matched (week 7) LGE MRI and histopathology slices were available and used for histopathological validation of dark‐blood LGE. Examples of in‐vivo LGE MRI and corresponding histopathology samples are shown in Fig. 3. Infarct size by dark‐blood LGE and conventional bright‐blood LGE correlated strongly with infarct size by histopathology (*r* = 0.998, *P* < 0.001 and *r* = 0.892, *P* < 0.001, respectively, Fig. 4a). Although Bland–Altman analyses demonstrated high levels of agreement for both comparisons with histopathology (Fig. 4b), conventional LGE showed a small but significant bias of −1.57% (*P* = 0.03). In contrast, dark‐blood LGE showed no significant bias when compared against histopathology (−0.03%, *P* = 0.75). The 95% limits of agreement were smaller for dark‐blood LGE (−0.90%, 0.84%) compared to conventional bright‐blood LGE (−8.02%, 4.88%). An example of conventional bright‐blood LGE and dark‐blood LGE imaging acquired at both 1‐week and 7‐weeks post‐MI is shown in Fig. 5.

### CNR Analysis

A combined total of 17 MRI slices with infarcted myocardium on both conventional bright‐blood and dark‐blood LGE, and available noise scan data, were available at 1‐week post‐MI, while a combined total of 26 MRI slices were available at 7‐weeks post‐MI.

#### ONE‐WEEK POST‐MI

**FIGURE 6:**
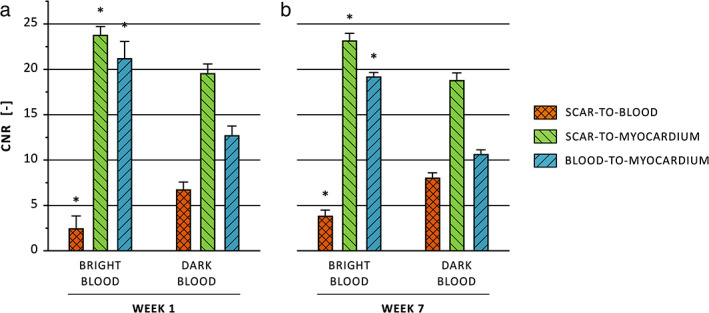
Contrast‐to‐noise ratios (CNRs) for conventional bright‐blood and dark‐blood LGE evaluated at 1‐week (**a**) and 7‐weeks (**b**) post‐MI. The scar‐to‐blood, scar‐to‐myocardium, and blood‐to‐myocardium CNRs were calculated by subtracting the SNRs of the two corresponding tissues. The error bars indicate the standard error of the mean. Asterisks indicate a significant difference between both methods (*P* < 0.05). MI = myocardial infarction; LGE = late gadolinium enhancement; SNRs = signal‐to‐noise ratios.

At 1‐week post‐MI, scar‐to‐blood contrast improved by 167% when using dark‐blood LGE compared to conventional bright‐blood LGE (6.86 vs. 2.57, *P* = 0.02, Fig. 6a). Scar‐to‐myocardium and blood‐to‐myocardium contrast decreased by 17.6% and 39.8%, respectively (19.7 vs. 23.9, *P* < 0.001 and 12.9 vs. 21.3, *P* < 0.001).

#### SEVEN‐WEEKS POST‐MI

At 7‐weeks post‐MI, a similar trend was observed as at 1‐week post‐MI. Dark‐blood LGE showed a significant increase of 106% in scar‐to‐blood contrast compared to conventional bright‐blood LGE (8.14 vs. 3.96, *P* < 0.001, Fig. 6b). Scar‐to‐myocardium and blood‐to‐myocardium contrast decreased by 18.8% and 44.3% when using dark‐blood instead of conventional LGE, respectively (18.9 vs. 23.3, *P* < 0.001 and 10.8 vs. 19.3, *P* < 0.001).

## Discussion

In this study, a novel dark‐blood LGE method, which does not require additional magnetization preparation, was validated against histopathology as reference standard in a porcine animal model.

Dark‐blood LGE showed excellent correlation and agreement with histopathology, with no significant bias. On the contrary, conventional bright‐blood LGE showed a significant bias of −1.6% (of the LV myocardium) in scar size, thereby underestimating the apparent scar tissue. Dark‐blood LGE showed smaller 95% limits of agreement compared to conventional LGE. Both at 1‐week and 7‐weeks post‐MI, dark‐blood LGE showed significantly improved scar‐to‐blood contrast compared to conventional LGE.

Dark‐blood LGE methods overcome an important limitation of conventional bright‐blood LGE. Since the blood pool and neighboring infarcted myocardium share nearly similar T_1_ relaxation times at 10 minutes following contrast media injection, both tissues often appear equally bright. Due to the resulting poor scar‐to‐blood, the apparent scar volume can be substantially underestimated or even completely obscured. In addition, determination of the scar‐blood barrier is challenging, hindering an accurate assessment of scar size and scar transmurality. Various novel methods were recently introduced to increase the scar‐to‐blood contrast using additional magnetization preparation mechanisms. The method validated in this study, however, does not use any additional magnetization preparation mechanism, making it readily and widely available in every clinical routine setting. By shortening the TI to the point of LV blood nulling, a darker appearance of the blood pool is achieved while maintaining the black appearance of normal myocardium and bright appearance of infarcted regions.

Although a large variety of dark‐blood LGE methods have been proposed, data on histopathological validation in animal models are scarce. In a recent study by Kim et al, a novel dark‐blood LGE method using magnetization transfer preparation, named “flow‐independent dark‐blood delayed enhancement” (FIDDLE), was evaluated in a canine animal model with histopathology as reference standard. FIDDLE showed excellent correlation and agreement with histopathology, with no significant bias. Conventional bright‐blood LGE, however, demonstrated a significant bias in infarct size of −1.1%. These findings are in line with the results of our study, where conventional bright‐blood LGE also underestimated infarct size by a similar extent while no significant bias was found for dark‐blood LGE. Though a few other studies performed dark‐blood LGE methods in animal models, none of these studies used histopathology as reference standard.^7^, ^8^, ^15^

The CNR analysis in this study demonstrated significantly higher scar‐to‐blood contrast for dark‐blood LGE compared to conventional LGE, both at 1‐week and 7‐weeks post‐MI. At 7‐weeks post‐MI, a significant increase of 106% in scar‐to‐blood contrast was observed, which is in line with findings of other recent studies.^8^, ^16^, ^19^ The small decrease in scar‐to‐myocardium contrast, when compared to conventional bright‐blood LGE, also agrees with other recent studies.^8^, ^9^, ^12^, ^16^

The increased scar‐to‐blood contrast achieved by dark‐blood LGE has been shown beneficial for the detection of small subendocardial scar regions.^12^, ^17^, ^20^ In addition, improved visualization of the scar‐blood barrier supports an accurate delineation of the scar region and directly influences scar size quantification.^12^ In the present study, the infarct regions included (near‐)transmural segments, in comparison to only subendocardial scar regions as mostly evaluated using other dark‐blood methods. Even though such transmural infarct regions are easily observed using conventional bright‐blood LGE, their quantification may still be hindered by the often‐vague scar‐blood barrier. The results of the present study support this theory, demonstrating a significant bias for conventional bright‐blood LGE when assessing the infarct size of these (near‐)transmural infarcts. Dark‐blood LGE therefore not only plays a role in detecting small subendocardial infarcts,^21^ but also in the quantification of larger (near‐)transmural infarct regions, which is crucial for clinical decision making (for instance benefit of revascularization).

In terms of efficiency and ease of use, dark‐blood LGE without additional magnetization is essentially identical to conventional bright‐blood LGE. All aspects regarding the acquisition, reconstruction, and contrast agent remain identical, and the same scan protocol can be used without any software modifications, parameter optimizations, or additional training. The sole difference between both methods is the TI selection. Instead of setting the TI for normal myocardium nulling in a standard PSIR sequence, the TI is set for LV blood pool nulling using the same standard Look‐Locker/TI scout scan. These properties guarantee easy implementation into clinic workflows, making dark‐blood LGE without additional magnetization preparation a readily available replacement method for conventional bright‐blood LGE. Additionally, with LGE MRI increasingly used in randomized trials as a measure of therapeutic efficacy for novel post‐MI treatments, dark‐blood LGE is expected to play an important role due to its increased accuracy and reduced variability in infarct size quantification.

### Limitations

There are limitations to this study that need to be addressed. As per study protocol, the animals underwent MRI examination twice and were sacrificed directly after the second MRI examination, at 7‐weeks post‐MI. Histopathological validation was therefore only available for the LGE images of the second MRI examination. In order to perform in‐vivo cardiac MRI at both 1‐week and 7‐weeks post‐MI, taking into account the increasing dimensions of the animals, imaging was performed at 1.5 T only due to the larger bore size available at our institution. For the same reason, in‐vivo MRI assessment of more chronic infarct regions beyond 7‐weeks post‐MI was limited.

Finally, the number of animals that underwent the entire protocol, including the second MRI examination and sacrifice required for histopathological validation, is fairly small. The majority of pigs that did not survive the entire protocol died within 4 hours post‐MI as a result of severe arrhythmias. The high vulnerability for arrhythmias post‐MI has been observed in other studies evaluating models of infarction in pigs.^22^ Even though this limited number, the authors feel that the present study outcomes would not have changed with an increased number of animals.

## Conclusion

Dark‐blood LGE without additional magnetization preparation provides superior visualization and quantification of ischemic scar patterns compared to the current in‐vivo reference standard. Infarct size as assessed by dark‐blood LGE demonstrated superior correlation and agreement compared to conventional bright‐blood LGE, using histopathology as reference standard. Since this dark‐blood LGE method does not require any additional magnetization preparation, it guarantees an easy and swift implementation in every clinical routine setting as a potential replacement for conventional bright‐blood LGE.
